# Rare extracranial localization of primary intracranial neoplasm

**DOI:** 10.1186/1746-1596-3-14

**Published:** 2008-04-16

**Authors:** Susan Arndt, Thorsten Wiech, Irina Mader, Antje Aschendorff, Wolfgang Maier

**Affiliations:** 1 Department of Oto-Rhino-Laryngology, Head and Neck Surgery University Medical Center Freiburg, Germany; 2 Department of Pathology, University Medical Center Freiburg, Germany; 3 Section of Neuroradiology, Neurocenter of the Freiburg University Hospital, Germany

## Abstract

Meningioma, craniopharyngeoma and glioma are mainly intracranial lesions. Nevertheless, in rare cases these entities may occur solely as extracranial lesions that may present as intranasal/sinusoidal masses, with headaches and nasal obstruction. We present three cases of common intracranial tumors, with purely extracranial extension. The three described cases demonstrate, that preoperative MRI and CT imaging is important for differential diagnosis to exclude intracranial connections of the tumors. A definitive diagnosis requires specialized immunohistochemical examinations. In all cases of intranasal or pharyngeal neoplasm the diagnosis of meningioma, craniopharyngeoma and glioma should be considered as differential diagnosis to optimize the surgical procedure.

## Background

Meningiomas, craniopharyngiomas and gliomas are mainly intracranial lesions. Nevertheless, in rare cases these entities may occur solely as extracranial lesions that may present as intranasal or sinusoidal masses, with vision disturbances, headaches and nasal obstruction. We present three cases of common intracranial tumors, with purely extracranial extension. In all patients, the tumor became manifest in symptoms resembling a primary extradural entity, like chronic sinusitis, chordoma or nasal polyposis. The true diagnosis is based mainly on histopathological examination.

## 1. Case report

**Figure 1:**
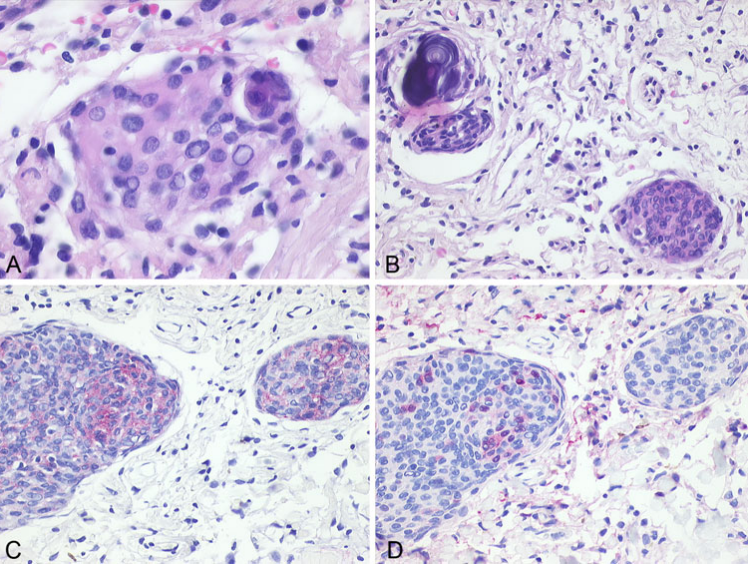
**Histological findings in meningothelial meningioma**. A: Lobule of tumor cells with oval nuclei and intranuclear inclusions (H&E 40×). B: Infrequently psammoma bodies were present (H&E 20×). C: Immunohistochemical detection of epithelial membrane antigen (EMA; 20×). D: Immunohistochemical detection of S100 (20×).

**Figure 2:**
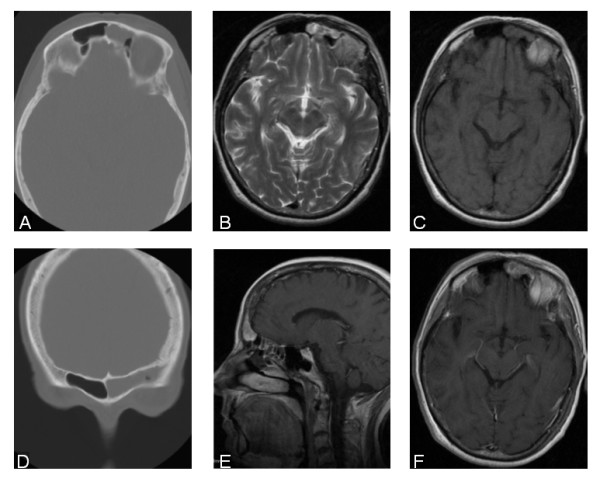
**Sinonasal meningeoma**. CT scan in bone window (A, D) reveals a soft tissue mass in the left frontal sinus without any signs of bone destruction or reaction. Axial T2 weighted images (B), T1 weighted images before (C) and after contrast administration (E, F) show a contrast enhancing soft tissue mass being slightly inhomogeneous in the left frontal sinus without any intracranial meningeal enhancement.

We report on a 60-year-old woman with swelling in the left periorbital region and frontal headache at the same side. Her medical history was significant for sinus surgery 9 months before elsewhere. General ENT examination showed no recurrent nasal polyposis. Contrast enhanced magnetic resonance imaging (MRI) revealed involvement of left ethmoid and frontal sinuses. Computed tomography (CT) scans confirmed the findings with the suspicion diagnosis of a frontal sinus mucocele. The patient underwent transfacial sinus surgery according to RITTER-JANSEN [1,2] performing a median drainage with placeholder for 3 weeks. The bony wall of the frontal sinus and the dura were unaffected. Histopathological examination showed lobules and fascicles of uniform partly spindle-shaped tumor cells with oval nuclei and intranuclear clear inclusions (Fig. 1). Infrequently, whorl formation and psammoma bodies were present. Cellular atypia, necrosis, or increased mitotic activity was not detected. Immunohistochemistry revealed weak expression of epithelial membrane antigen (EMA), vimentin and S-100. The histopathological diagnosis was a meningothelial meningioma corresponding to WHO grade I [3]. There was no evidence of a connection of this sinonasal meningioma to other intracranial masses, as indicated in CT scan in bone window and MR imaging (Fig. 2).

## 2. Case report

A 16-year-old girl presented to our department with nasal obstruction and headache in the occipital region. Her medical history was significant for two turbinoplasties within two months in the last year elsewhere with persistency of the disturbances. General ENT examination showed a soft tumor in the nasopharynx. The overlying mucosa was normal in appearance.

**Figure 3:**
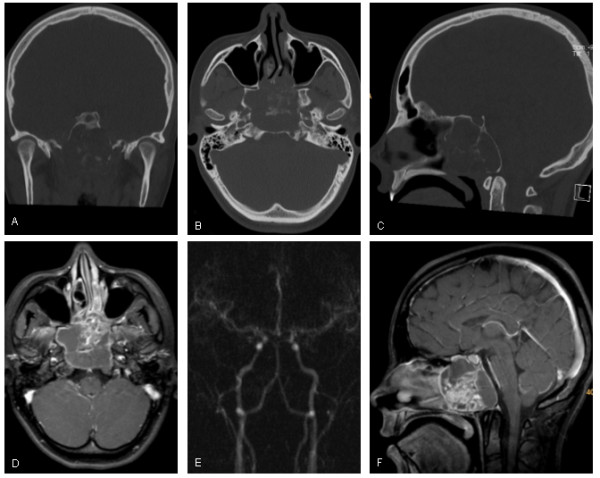
**Extracranial craniopharyngeoma**. A-C: Coronal, axial and sagittal reconstructions of a spiral CT scan. Note the expansively growing mass with calcifications, thinning and remodeling of the walls of the pterygoid processes, predominantly on the right side, and the anterior parts of the clivus with preserved posterior margin. D and F: Axial and sagittal contrast-enhanced T1-weighted images from a 3D data revealing a partly solid and cystic mass of the clivus and sphenoid cavity with expansion into the ventral the ethmoid sinuses and the nasal cavity, caudally limited by the plane of soft and hard palate and cranially by the sella. E: Early arterial phase of a time resolved contrast-enhanced MR angiography to exclude an juvenile angiofibroma. Neither pathological arterial vessels nor arteriovenous shunts were visible.

Radiological examinations included contrast enhanced CT- and MRI scans. CT scans demonstrated a well-defined expansive growing mass with a diameter of 5 cm comprising areas of calcifications and thinning and remodeling of the walls of predominantly the right pterygoid processes and clivus, but with preserved posterior and upper margin. Contrast-enhanced T1-weighted MRI showed a multilobulated, partly solid and cystic mass with central calcifications of the sphenoid cavity and clivus reaching both internal carotid arteries, and invading the ethmoid sinuses and the nasal cavity. The caudal border was the plane of soft and hard palate and the cranial border was the sella turcica. Time resolved contrast-enhanced MR angiography did not reveal any tumor blush or arteriovenoud fistulae (Fig 3).

The patient underwent transnasal endoscopic biopsy that showed adamantinomatous craniopharyngioma with focal keratinization, corresponding to WHO grade I [3]. Navigated microscopic/endoscopic sphenoidectomy and removal of the tumor from clivus and sellar floor with midfacial degloving was performed. The entire tumor was extradural. The tumor could be resected completely, safety margins were free of disease.

**Figure 4:**
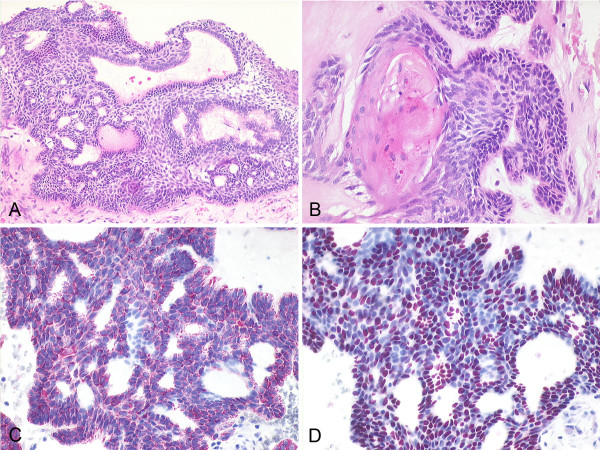
**Histological features of adamantinomatous craniopharyngioma with focal keratinization**. A: Bridging cords of tumor cells with palisading of basal nuclei (H&E 10×). B: Enclosed keratin nodule (H&E 20×). C: Immunohistochemical detection of high-molecular weight cytokeratin (20×). D: Nuclear expression of p63 (20×).

Histopathological examination including immunohistochemistry was performed of the tumor specimens. The tumor cells consisted were arranged in cords and bridges of multistratified epithelium with palisading of basal nuclei (Fig. 4). Nodules of compact keratin were frequently found. Immunohistochemistry revealed expression of pancytokeratin, high-molecular weight cytokeratin and p63. Additionally an expression of somatostatin receptor was detected.

## 3. Case report

**Figure 5:**
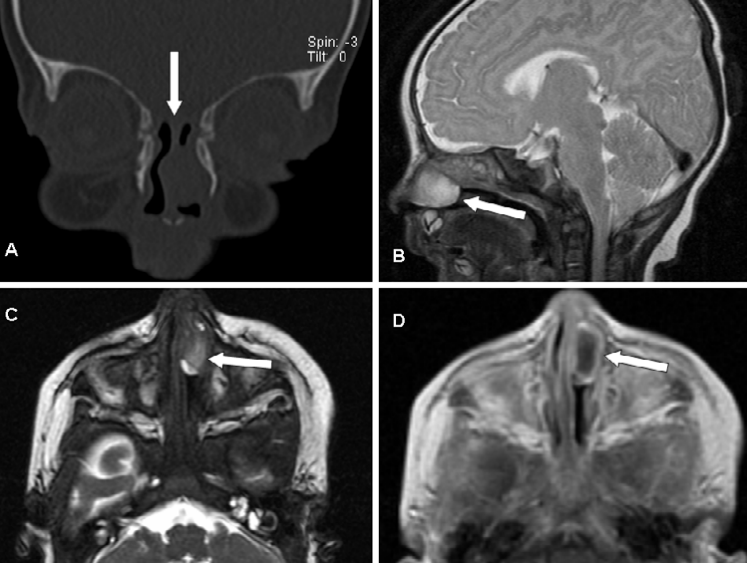
**Nasal glial heterotopia**. A: Coronal reconstruction of a spiral CT scan. A soft tissue mass is visible in left nasal cavity. Note the incomplete ossification of the skull base (arrow) at the age of 48 days. Ossification is regularly completed in this area at the age of 3 months. B: Sagittal T2 weighted MR image revealing an endonasal hyperintense mass without intracranial connection. C and D: axial T2 weighted and contrast-enhanced T1 weighted MR images showing a not contrast-enhancing mass in the nasal cavity. The rim-like contrast enhancement around the tumor is attributed to peritumoral mucosal swelling.

**Figure 6:**
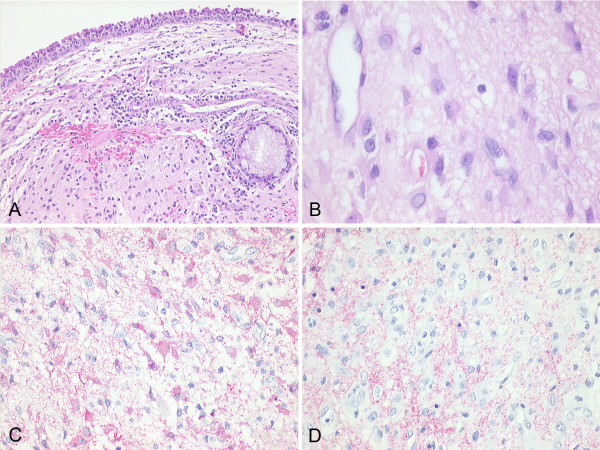
**Histological findings of endonasal glial heterotopia**. A: Subepithelial neuroglial tissue (H&E 10×). B: Astrocytes and capillaries embedded in a loose fibrillary matrix (H&E 40×). C: Immunohistochemical detection of glial fibrillary acidic protein (GFAP 20×). D: Focal immunohistochemical detection of synaptophysin (20×).

A 48-day-old boy was presented with polypous mass in the left nasal cavity with resultant breathing and feeding difficulties. The preoperatively performed MRI- and CT scans showed an endonasal soft-tissue mass without intracranial connection and according to the patient's age a not-yet ossified skull base (Fig. 5). The mass was removed in endoscopic surgery. No connection to the central nervous system or any cerebrospinal fluid (CSF) leak was found. Histological and immunohistochemical examination revealed neuroglial and fibrovascular tissue containing mature astrocytes with expression of glial fibrillary acidic protein (GFAP). In addition, immunoreactivity for vimentin, S-100, and focal expression of synaptophysin could be detected (Fig. 6). These features led to the diagnosis of endonasal glial heterotopia. The postoperative course was good, the breathing and feeding without abnormalities.

## Discussion

The three reports demonstrated that in all cases of intranasal or pharyngeal neoplasm the diagnosis of meningioma, craniopharyngioma and glioma should be considered as differential diagnosis. These three described tumors rarely appear in an extracranial localization.

Intracranial meningiomas are the most common adult benign intracranial neoplasms, whereas extracranial meningiomas are rare tumors comprising 1–2% of all meningiomas [4,5]. The prognosis of menigiomas is generally favorable. However, in rare cases meningiomas are aggressive and can occur as a malignant meningioma. Avninder [6] described a case of a papillary meningioma as an aggressive histological variant, which accounts for 1.0–2.5% of all meningiomas.

Extracranial sinonasal tract meningiomas often demonstrate an erosion of the sinus wall, with extension to the surrounding soft tissue, to the orbit and occasionally to the skull base. Complete surgical extirpation of sinonasal meningioma is the treatment of choice without the need for adjuvant treatment. Relapses are possible in case of incomplete removal of the primary formation. The prognosis of extracranial meningioma is always good if the excision is complete [7].

Craniopharyngiomas are benign but aggressive tumors deriving from cell rests from the Rathke's pouch and account for about 3% of all intracranial tumors [8,9]. Usually they are localized in the suprasellar region. The isolated infracranial localization was first described by Bock in 1924 [10]. Infrasellar craniopharyngiomas are exceedingly rare because the sphenoid bone imposes a limitation on caudal tumor expansion. Less than 10 cases of infrasellar craniopharyngiomas in which the tumor had no sella involvement have been described [11]. In contrast to patients with suprasellar craniopharyngiomas, generally presenting with headache and visual disturbance, patients with infrasellar craniopharyngiomas present usually only with nasal obstruction like in the present case. Surgical treatment of these tumors is indispensable. The approach is determined by the anatomic location of the tumor. Entirely infrasellar craniopharyngiomas may be removed completely, possibly offering a better prognosis than suprasellar craniopharyngiomas.

Nasal glial heterotopia are rare tumors, might derive from either separated neuroectodermal tissue during the closure of the covering brain, or from a nasal encephalocele which is covered by dura, pia, and arachnoid and later disconnected from the intracranial cavity during subsequent development. They manifest usually at birth or during early childhood and can cause a visible deformation, a nasal obstruction or chronic otitis media. Nasal gliomas may occur in extrananasal (60%) or intranasal localization (30%) or combined (10%) [12]. The treatment of choice is complete surgical excision to avoid deformations of immature facial bones, cartilage necrosis as well as infections. A biopsy should not be performed because of the risk of provoking menigitis or injuring intact brain tissue. The overall outcome is good, depending on complete excision. Recurrences occur in 4–10%, most likely due to incomplete primary resection [13].

## Conclusion

All three present cases demonstrate that preoperative MRI and CT imaging is important for differential diagnosis to exclude intracranial connections of the tumor. A definitive diagnosis requires histopathological and immunohistochemical examination. In all cases of intranasal or pharyngeal neoplasm the diagnosis of meningioma, craniopharyngeoma and glioma should be considered as a differential diagnosis, and intracranial connections should be excluded to optimize the surgical procedure.

## Competing interests

The author(s) declare that they have no competing interests.

## Authors' contributions

SA and AA drafted the manuscript, TW evaluated the immunohistochemical stainings and confirmed the diagnoses, SA, WM and AA performed the surgeries and compiled the clinical data. IM evaluated the radiological diagnostics, SA and WM mainly contributed to the discussion. All authors read and approved the final manuscript.

## References

[B1] Ritter G (1906). Eine neue Methode zur Erhaltung der vorderen Stirnhöhlenwand bei Radikaloperationen chronischer Stirnhöhleneiterungen. Dtsch Med Wochenschr.

[B2] Jansen A (1894). Zur Eröffnung der Nebenhöhlen der Nase bei chronischer Eiterung. ArchLaryngol Rhinol.

[B3] Kleihues P, Cavenee WK, (eds) (2000). Pathology and genetics of tumours of the nervous system.

[B4] Ismail H, Burnley H, Harries PG (2004). Recurrent extracranial sinonasal meningioma presenting 27 years after complete surgical eradication of right frontal meningioma. Acta Otolaryngol.

[B5] Lusis E, Gutmann DH (2004). Meningioma: an update. Curr Opin Neurol.

[B6] Avninder S, Vermani S, Shruti S, Chand K (2007). Papillary meningioma: a rare but distinct variant of malignant meningioma. Diagn Pathol.

[B7] Kumar S, Dhingra PL, Gondal R (1993). Ectopic meningioma of the paranasal sinuses. Childs Nerv Syst.

[B8] Russell D, Rubinstein L (1989). Pathology of tumors of the nervous system.

[B9] Yamini B, Narayanan M (2006). Craniopharyngiomas: an update. Expert Rev Anticancer Ther.

[B10] Bock E (1924). Beitrag zur Pathologie der Hypophyse, Virchow. Arch Pathol Anat.

[B11] Deutsch H, Kothbauer K, Persky M, Epstein FJ, Jallo GI (2001). Infrasellar craniopharyngiomas: Case report and review of the literature. Scull base.

[B12] Penner CR, Thompson L (2003). Nasal glial heterotopia: a clinicopathologic and immunophenotypic analysis of 10 cases with a review of the literature. Ann Diagn Pathol.

[B13] Dasgupta NR, Bentz ML (2003). Nasal gliomas: identification and differentiation from hemangiomas. J Craniofac Surg.

